# Editorial: Biosafety and Biosecurity Approaches to Counter SARS-CoV-2: From Detection to Best Practices and Risk Assessments

**DOI:** 10.3389/fbioe.2021.752909

**Published:** 2021-08-24

**Authors:** Segaran P. Pillai, Jianming Qiu, Stephen A. Morse

**Affiliations:** ^1^ Office of the Commissioner, United States Food and Drug Administration, Silver Spring, MD, United States; ^2^ Department of Microbiology, University of Kansas Medical Center, Kansas City, KS, United States; ^3^ IHRC, Inc, Atlanta, GA, United States

**Keywords:** biosafety and biosecurity, SARS-CoV2, detection, best practices, risk assessments, diagnostics, PPE

Scientific communications are important for addressing technical issues that can impact the COVID-19 pandemic. To this end, Frontiers developed a Research Topic entitled “Biosafety and Biosecurity Approaches to Counter SARS-CoV-2: From Detection to Best Practices and Risk Assessment.” Thirty-four manuscripts from 14 countries were originally submitted for this Research Topic. Of these, 18 (53%) were accepted. The 18 accepted papers that comprise this Research Topic were originally submitted to three Frontiers journals: Bioengineering and Biotechnology (*N* = 9), Medicine (*N* = 6), and Public Health (*N* = 3). The types of papers consist of original research articles (*N* = 7), brief research reports (*N* = 4), methods articles (*N* = 1), opinions (*N* = 2), and review articles (*N* = 4). The ten countries from which the accepted manuscripts were submitted truly represents the scope of the pandemic: United States (*N* = 5), China (*N* = 4), and 1 each from France, Lebanon, Panama, Russia, Mexico, Bahrain, Spain, Portugal, and Greece.

When this Research Topic began, there were many unanswered questions including the origin of the novel SARS-CoV-2, its pathogenicity, transmissibility, efficacy of existing medical countermeasures and supportive therapies, and its survival in the environment. An et al. reviewed recent progress in the field of synthetic biology and the laws and regulations governing its use to avoid potential risks associated with this technology. As with other infectious agents, the environment can play an important role in the transmission of SARS-CoV-2. In an innovative on-line forum, Morrow et al. reviewed the challenges that industries from around the globe experienced in reducing the transmission of this virus in an indoor environment. Based on the results, the authors called for significant investments in research to understand virus persistence and transport in the built environment.

Buhr et al. used the enveloped RNA bacteriophage φ6 as a surrogate for SARS-CoV-2 virus to study its inactivation in an aircraft environment. The surrogate was dried on wiring insulation, aircraft performance coating, polypropylene and nylon at >8log10 PFU/test coupon. Modeling showed that a 1-h treatment of a C-130 aircraft with hot (≥63°C) humid (90% RH) air had a 90% probability of inactivating the virus by >7 log10.

The extent of the pandemic has exacerbated the availability of critical supplies such as PPE. Two papers proposed strategies to overcome the shortage of PPE. Bernard et al. described a proof-of-concept study to address the shortage of surgical masks and N95 filtering face-piece (FFP) two respirators. They demonstrated that treating used surgical masks and FFP2 respirators in a chamber for 1 h at 70°C and 75% RH was sufficient to kill surrogate bacteria and viruses while maintaining the filtering capacity of the PPE. Thus, reusing used PPE during mass shortfalls is possible. In the other paper, Kothakonda et al. applied open-source product development to develop locally manufactured, modular, powered air-purifying respirator (PAPR) components, including filter cartridges and blower units. Two designs, one with a fully custom-made filter and blower unit housing, and the other with commercially available variants were developed. Engineering testing and clinical feedback demonstrated that the designs represented favorable alternative PAPRs for use during shortfalls that may occur during pandemics.

Hospitals can be a source of new infections. To reduce this possibility, Raventos and Sabata suggested that air curtains equipped with sprayers to nebulize an alcoholic solution could be used in hospitals to disinfect clothing, exposed body parts and objects that passed through to rapidly and economically reduce the propagation of the virus. Hospital workers caring for COVID-19 patients are of increased risk of acquiring SARS-CoV-2. In a study, Wang et al. reported that the use of an oropharyngeal probiotic *Streptococcus thermophilus* ENT-K-12, in a slow-dissolving lozenge form, twice a day, to create a stable upper respiratory tract microbiota, significantly reduced the incidence of certain respiratory tract infections [22/95 (23.2%) control group vs 8/98 (8.2%) probiotic group, *p* = 0.004] among front-line physicians and nurses attending COVID-19 patients in a hospital setting.

In today’s world, the requirement for genetic information security is an ever-growing need. The very information that allows us to understand the properties of a pathogen can also be misused through genetic manipulation for potential harm. Schumacher et al. characterized a general genetic information system from biological material collection through long-term data sharing, storage, and application in the context of security. They also discussed the challenges associated with wet and dry laboratories due to distributed devices and systems that are not designed to address the security of genetic information systems and the need for an extensive laboratory system to realize the potential of this emerging field and to protect the bioeconomy of all stakeholders.

Habli et al. reviewed the current state-of-the-art of point-of-care diagnostic platforms for the rapid detection of COVID-19 and its seroprevalence throughout the cycle of infection. The review analyzed their performance characteristics and discussed limitations with respect to COVID-19. Because of the infectiousness of SARS-CoV-2, package inserts from antibody detection kits recommended that serum samples be heat inactivated before analysis. However, Lin et al. observed that heat-inactivation significantly increased values for SARS-CoV-2 IgG antibody while values for SARS-CoV-2 IgM antibody decreased with increasing temperature of heat inactivation. This effect was dependent upon the method used for antibody detection, which pointed out the necessity for laboratories to evaluate the kits to ensure accurate COVID-19 detection results.

Villarreal et al. evaluated a lateral flow assay (LFA) that detects both IgG and IgM in serum samples from: 1) COVID-19 patients with a confirmed positive RT-PCR; 2) potentially exposed healthcare workers; and 3) healthy blood donors. The LFA had a positive percent agreement of 97.2%. The evaluation of serum samples from hospitalized COVID-19 patients indicated a correlation between test sensitivity and the number of days since onset of symptoms. The seroprevalence among healthcare workers who reported close contact with confirmed COVID-19 patients was 12.9% versus 1.8% among those who did not report close contact.

Varlamov et al. evaluated a combination of polymerase chain reaction (PCR) and isothermal nucleic acid amplification techniques, which included conventional PCR and loop-mediated isothermal amplification (LAMP) methods, with hybrid techniques such as polymerase chain displacement reaction (PCDR) and a newly developed PCR-LAMP method. Based on their evaluation, they concluded that hybrid methods exhibited higher sensitivity and assay reaction rates than the classic LAMP- and PCR-based methods.

To ascertain early warning signs for severe and critical patients with COVID-19, Xu et al. performed a quantitative analysis of chest CT images at the lung segment level. Their analysis showed that lung involvement in the ordinary and severe/critical groups reached a peak on the 18th and 14th day, respectively. In the first stage, the percentage of lung involvement (PLI) in the right middle lobe and the left superior lobe were significantly different between the two groups. In the second stage and the fourth stage, there were statistically significant differences in PLI between the two groups in the whole lung, right superior lobe, right inferior lobe and left superior lobe. They observed that the rapid progress of the lateral segment of the right middle lobe on the second day and the anterior segment of the right upper lobe on the 13th day may be an early warning sign for severe/critical patients.

Sample type and sample processing are critical for the rapid, sensitive, and accurate diagnosis of infections such as COVID-19. Rodriguez-Flores et al. compared results of nasopharyngeal swabs and saliva samples from the same COVID-19 patients using a standard nucleic acid extraction protocol including protein lysis with proteinase K followed by binding to a column, washing, and elution, with the SalivaDirect protocol based on protein lysis and skipping the other steps to reduce processing time and cost. They noted that with the SalivaDirect protocol, saliva samples had a diagnostic sensitivity of 88.2% whereas nasopharyngeal swab samples had a diagnostic sensitivity of 93.6%.

An understanding of viral clearance in asymptomatic SARS CoV-2-infected individuals is critical for the development of interventions to minimize transmission and for public health messaging. Kumar et al. studied the timeline for viral clearance in 145 asymptomatic and 39 non-clinical symptomatic individuals. Based on their analysis, the median time till viral negativity for subclinical and for overt infections was 11 days after controlling for age and sex.

Batiha et al. have written a review article on macrolides such as azithromycin, as well as chloroquine/hydroxychloroquine, and proposals that have been made regarding their potential for consideration in COVID-19. Readers should be aware that the COVID-19 Treatment Guidelines on the Unites States NIH website recommend against use of chloroquine or hydroxychloroquine and/or azithromycin for treatment of COVID-19 (https://www.covid19treatmentguidelines.nih.gov/therapies/antiviral-therapy/chloroquine-or-hydroxychloroquine-and-or-azithromycin/).

Li et al. adopted a deep learning model to predict fatality of individuals that tested positive given the patient’s underlying health conditions, age, sex, and other factors. As the allocation of resources towards a vulnerable patient could mean the difference between life and death, a fatality prediction model may serve as a valuable tool in prioritizing resources and hospital space.

While most children infected with SARS-CoV-2 are asymptomatic or develop mild symptoms, some have been hospitalized and some have died. Feketea and Vlacha developed an algorithm called “STUDY SAFE” that when used together with telemedicine can help parents decide when to test a symptomatic low risk child, and if positive, when the child can return to school.

In addition to the contribution of these scientists toward our understanding of COVID-19 response, scientists from around the world are working to enhance our knowledge and understanding about this virus and trying to identify ways to counter and eradicate this disease. Even with all of our current understanding, many unanswered questions remain that will be worth exploring. The editors of this Research Topic would like to thank the many scientists for their contribution to our knowledge about this disease and their dedication to public health.

## Author Disclaimer:

This editorial summarizes papers submitted to our Research Topic and is not an endorsement by the authors’ agency or institution of the view or position of the paper’s authors.

